# Targeting the EGF receptor in ovarian cancer with the tyrosine kinase inhibitor ZD 1839 (‘Iressa’)

**DOI:** 10.1038/sj.bjc.6600058

**Published:** 2002-02-01

**Authors:** J M Sewell, K G Macleod, A Ritchie, J F Smyth, S P Langdon

**Affiliations:** Imperial Cancer Research Fund Medical Oncology Unit, Western General Hospital, Edinburgh EH4 2XU, UK

**Keywords:** tyrosine kinase inhibitor, ovarian cancer, EGF receptor, ZD 1839, Iressa

## Abstract

The modulating effects of the orally active epidermal growth factor receptor-specific tyrosine kinase inhibitor ZD 1839 (‘Iressa’) on cell growth and signalling were evaluated in four ovarian cancer cell lines (PE01, PE04, SKOV-3, OVCAR-5) that express the epidermal growth factor receptor, and in A2780, which is epidermal growth factor receptor-negative. Transforming growth factor-α stimulated growth was completely inhibited by concentrations of ZD 1839 ⩾0.3 μM in the epidermal growth factor receptor-expressing cell lines, as were transforming growth factor-α stimulated phosphorylation of the epidermal growth factor receptor and downstream components of the MAP kinase and PI-3 kinase signalling cascades. Growth inhibition in the absence of added transforming growth factor-α was also observed which could be consistent with suppression of action of autocrine epidermal growth factor receptor-activating ligands by ZD 1839. In support of this, transforming growth factor-α, EGF and amphiregulin mRNAs were detected by RT–PCR in the epidermal growth factor receptor-expressing cell lines. ZD 1839 inhibited growth of the PE04 ovarian cancer xenograft at 200 mg kg^−1^ day^−1^. These data lend further support to the view that targeting the epidermal growth factor receptor in ovarian cancer could have therapeutic benefit.

*British Journal of Cancer* (2002) **86**, 456–462. DOI: 10.1038/sj/bjc/6600058 www.bjcancer.com

© 2002 The Cancer Research Campaign

## 

The epidermal growth factor receptor (EGFR) is a 170 kDa transmembrane glycoprotein that contains an external binding domain and an intracellular tyrosine kinase domain. It mediates the mitogenic response to the EGF family of ligands which includes both EGF and transforming growth factor-α (TGFα) (Carpenter, 1987). Ligand binding activates the EGFR by inducing either homodimerization or heterodimerization with other members of the ErbB receptor family (ErbB2, ErbB3 and ErbB4) (Olayioye *et al*, 2000). Upon dimerization, key autophosphorylation events occur on C-terminal tyrosine residues of the intracellular domain which provide high-affinity docking sites for a series of transducing molecules that transmit the mitogenic signal to the nucleus. Signalling cascades such as the Ras/Raf/ERK/MAPK pathway (Egan and Weinberg, 1993), PI-3K/AKT pathway (Verbeek *et al*, 1998) and the PLCγ pathway (Chen *et al*, 1994) have all been implicated as routes to gene activation or more direct modulators of mitogenesis and other cancer-promoting phenotypes.

The EGFR is reported to be present in between 33 and 75% of ovarian cancers (Berchuck *et al*, 1991; Morishige *et al*, 1991) and has been implicated in both the growth and progression of this disease. Ovarian cancers that express increased concentrations of the EGF receptor are associated with poor survival (Berchuck *et al*, 1991; Bartlett *et al*, 1996) and both TGFα and EGF have been shown to stimulate growth of ovarian cancer cells in culture (Morishige *et al*, 1991; Crew *et al*, 1992). Studies utilizing NIH 3T3 cells have indicated that overexpression of EGFR in the presence of a stimulatory ligand is sufficient to confer cancer phenotypes (Verbeek *et al*, 1998). Evidence for both autocrine and paracrine regulation of growth by TGFα/EGFR activation has been obtained in this disease (Morishige *et al*, 1991; Stromberg *et al*, 1992).

Given the importance of this receptor in both ovarian cancer growth and progression, it represents a good target for anticancer drug development. A variety of strategies to block activation of the receptor have been developed and these include use of anti-EGFR blocking monoclonal antibodies and EGFR-targeted tyrosine kinase inhibitors.

In the present study we have explored the potential of a new EGFR targeted tyrosine kinase inhibitor (ZD 1839, ‘Iressa’) to inhibit growth of ovarian cancer. Enzymatic activity of the intracellular tyrosine kinase domain of the EGFR is essential for signal transduction (Chen *et al*, 1987) and a number of specific and potent inhibitors of the EGF receptor have been identified (Fry *et al*, 1994; Levitzki and Gazit, 1995; Wakeling *et al*, 1996). We have previously demonstrated that one of these inhibitors, ZM 252868 (PD 153035, 4(3-bromoanilino)-6,7-dimethoxyquinazoline) inhibited ovarian cancer cell growth and tyrosine phosphorylation on the EGFR (Simpson *et al*, 1999), however this compound was ineffective *in vivo* (Kunkel *et al*, 1995). A related structure, ZD 1839, has been developed which is not only a potent and selective inhibitor of the EGFR tyrosine kinase but has demonstrated excellent growth inhibitory activity after oral administration (Woodburn *et al*, 1997; Ciardiello *et al*, 2000). The drug is currently under clinical evaluation in several Phase I–III trials in cancer patients (Baselga *et al*, 2000; Ferry *et al*, 2000). In the present study we have tested its activity against five ovarian cancer cell lines *in vitro* to assess its growth inhibitory activity and its ability to inhibit TGFα activated EGFR phosphorylation in this disease. We have also assessed its ability to inhibit growth of an ovarian cancer xenograft growing in nude mice.

## MATERIALS AND METHODS

### Cell lines

The human ovarian carcinoma cell lines used were; PE01 and PE04 (Langdon *et al*, 1988), SKOV-3 and A2780 (European Collection of Animal Cell Cultures, Porton Down, UK), and OVCAR-5 (kindly provided by Dr T Hamilton, Fox Chase Institute, PA, USA). All lines were routinely cultured at 37°C in a humidified atmosphere of 5% CO_2_ in air, using ‘full media’; RPMI 1640 containing phenol red (Gibco BRL, Paisley, UK) supplemented with 10% heat-inactivated foetal calf serum (FCS), L-glutamine (2 mM), penicillin (100 IU ml^−1^) and streptomycin (100 μg ml^−1^).

### Growth assays

Log-phase cells were trypsinized and seeded in full media into 24-well plates (Falcon) in quadruplicate at densities between 0.5–2.5×10^4^ cells per well^−1^. After 24 h media was replaced (following two phosphate-buffered saline (PBS) washes) with phenol red free RPMI 1640 (PRF-RPMI) containing 5% double charcoal-stripped foetal calf serum, penicillin (100 U ml^−1^), streptomycin (100 μg ml^−1^), glutamine (2 mM). After a further 24 h, drugs and fresh media were added to cells in PRF-RPMI 1640; designated day 0. Tyrosine kinase inhibitor ZD 1839 was added 30 min prior to the addition of TGFα (1 nM). Drugs and media were replaced on day 2, then cells harvested on day 5 and counted using a Coulter counter (Coulter Electronics Ltd., Luton, UK).

### Western blotting

Cell lines were seeded in full media into 60 cm^2^ petri dishes (Nunclon). After 24 h, cells were washed twice with PBS before adding PRF-RPMI. A further 24 h later, (cells 60–80% confluent) plates were treated for 30 min with PRF-RPMI media in the presence or absence of ZD 1839 (0.03–3.0 μM). Media ±ZD 1839 was then replenished and incubated for a further 15 min in the presence or absence of TGFα (1 nM). Cells were washed twice in ice cold PBS, and lysed using 1 ml hypotonic lysis buffer (50 mM Tris-HCl (pH 7.5), 5 mM EGTA (pH 8.5), 150 mM NaCl, 1% Triton X-100, 2 mM sodium orthovanadate, 50 mM sodium fluoride, 20 μM phenylarsine oxide, 1 mM phenylmethanesulfonylfluoride, 10 μg ml^−1^ leupeptin, 10 μg ml^−1^ aprotinin and 10 mM sodium molybdate). Lysates were clarified by centrifugation for 6 min at 13 000 r.p.m. in a microfuge at 4°C. Total protein concentrations of supernatants were determined using a Bio-rad protein Assay Kit (Bio-rad, Richmond, CA, USA).

Samples (35–75 μg) were denatured at 95°C for 5 min in buffer containing SDS and mercaptoethanol and resolved by 7.5% SDS–PAGE. They were transferred onto Immobilon-P membranes (Millipore, Bedford, MA, USA) overnight at 4°C. After transfer, membranes were blocked with 1% blocking reagent (containing 10% purified casein protein in maleic acid, Boehringer Mannheim) diluted in TBS (Tris buffered saline; 20 mM Tris-HCl, 137 mM NaCl, pH 7.5) for 1 h at room temperature. Primary antibodies were added in 0.5% TBS/blocking solution and left overnight at 4°C; EGFR (Neomarkers Ab12) 1/200, phosphotyrosine (Santa Cruz PY20) 1/200, phospho-ERK (NEB 9101) 1/1000 and phospho-AKT^S473^ (NEB 9271) 1/1000. Membranes were then washed; 3×5 min TBS-T (TBS with 0.1% Tween 20), and 2×5 min in 0.5% TBS/blocking solution, then incubated with appropriate secondary antibody for 1 h at room temperature. Membranes were washed again; 3×5 min with TBS-T, 3×5 min with TBS. After 1 min incubation in room temperature luminescence substrate solution, light emission was detected on X-ray film.

### RT*–*PCR

Total cellular RNA was extracted from cells in log phase growth using TRI reagent™ (Sigma, Poole, UK). Samples were treated with 20 U/50 μl DNAse 1 (Boehringer Mannheim, East Sussex, UK) to remove genomic DNA contamination. RNA was then re-extracted using a phenol/chloroform protocol. Reverse transcription was performed with a first strand cDNA Synthesis kit (Boehringer Mannheim) using the oligo dT primer provided. RNA (1 μg) yielded 20 μl of cDNA of which 2 μl was used for each subsequent PCR reaction with each primer pair (Table 1

**Table 1:**
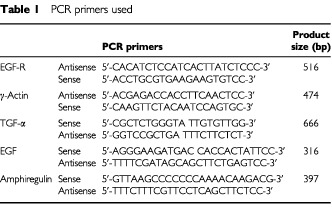
PCR primers used

). PCR reactions (20 μl) contained: 1×buffer, 1.5 mM MgCl_2_, 0.2 mM dNTP mixture, 1.0 U Taq polymerase (ICRF, Clare Hall, South Mimms, UK), 500 nM each primer (ICRF). RNA which had not been reverse transcribed was used in PCR reactions to check for genomic contamination of RNA samples.

### Xenograft experiments

The PE04 xenograft was initiated from the cultured PE04 ovarian cancer cell line and maintained subcutaneously in the flanks of female nude mice. Animals were at least 8 weeks old at the time of experimentation and were maintained in negative pressure isolators (La Calhene, UK). For experiments, tumour fragments were implanted and allowed to develop to 4–6 mM (period of approximately 1 month). Animals were then allocated to treatment (five mice per group) or control (10 mice per group) groups and treatment commenced (defined as day 0).

Treatment groups contained 8–10 tumours and control groups 14–18 tumours. The drug was given orally as a suspension in 0.5% polyoxyethylenesorbitan (Tween 80) for 14 days. Tumour size was measured twice weekly using calipers and the volume calculated according to the formula: π/6×length×width^2^. Relative tumour volumes were then calculated for each individual tumour by dividing the tumour volume on day t (V_t_) by the tumour volume on day 0 (V_0_) and multiplying by 100. Body weight changes were monitored every 4 days to monitor toxicity. All experiments were conducted according to UKCCCR (1998) guidelines under a Home Office licence.

### Statistical analysis

The Student *t*-test was used to compare differences between groups.

## RESULTS

### The tyrosine kinase inhibitor ZD 1839 inhibits TGF*α* stimulated growth of ovarian cancer cells* in vitro*

**Figure 1:**
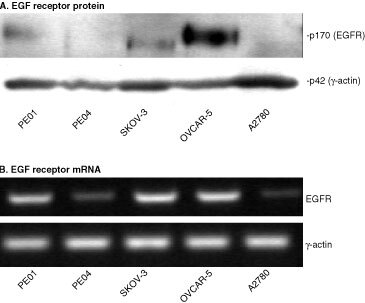
Western blot and RT–PCR analyses of the EGFR in ovarian cancer cell lines. The PE01, PE04, SKOV-3, OVCAR-5 and A2780 ovarian cancer cell lines were subjected to (**A**) Western blot analysis using antibodies targeting the EGFR and actin or (**B**) RT–PCR using primers specific for either the EGFR or γ-actin as described in Materials and Methods.

**Figure 2:**
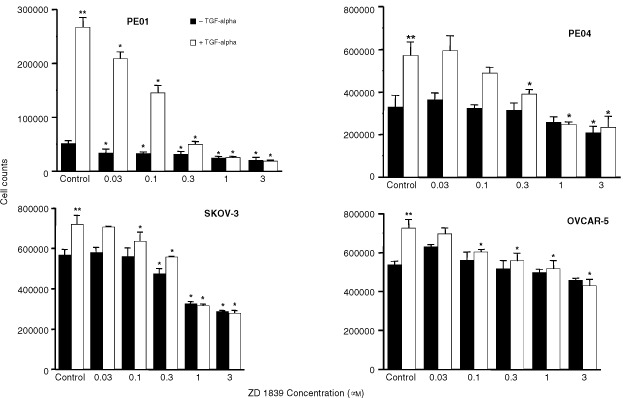
Effect of ZD 1839 on the basal and TGFα-stimulated growth of the PE01, PE04, SKOV-3 and OVCAR-5 ovarian cancer cell lines. ZD 1839 added in the absence or presence of TGFα (1 nM). Statistically significant differences: ** control *vs* control + TGFα=*P*<0.05; Student *t*-test ; *test *vs* appropriate control=*P*<0.05, Student *t*-test.

**Figure 3:**
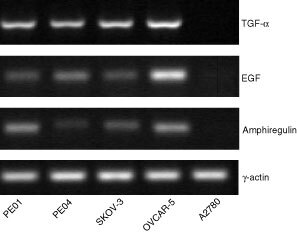
RT–PCR analysis of EGFR activating ligands in ovarian cancer cell lines. mRNAs from the PE01, PE04, SKOV-3, OVCAR-5 and A2780 cell lines were reverse transcribed. Use of specific primers for TGFα, EGF, amphiregulin and γ-actin allowed specific amplification of these cDNAs as described in Materials and Methods.

Five ovarian cancer cell lines (PE01, PE04, SKOV-3, OVCAR-5 and A2780) were investigated for their response to the EGFR-targeted inhibitor ZD 1839. Expression levels of EGFR vary widely in this series of cell lines, with A2780 indicating a very weak EGFR mRNA band and no visible protein, PE04 and PE01 expressing only low levels, SKOV-3 intermediate levels and OVCAR-5 the highest level of EGFRs (Figure 1

). We have previously demonstrated expression of the EGF receptor in PE01 and PE04 cells using ligand binding and immunofluorescence techniques (Crew *et al*, 1992). The five cell lines were treated with ZD 1839 for 5 days in the presence or absence of 1 nm TGFα and growth response was assessed. Addition of 1 nM TGFα produced an approximately 450% increase in PE01 cell number, a 67% increase for PE04 and an increase of 20–30% in the SKOV-3 and OVCAR-5 cell lines. This increase was abolished by ZD 1839 at concentrations ⩾0.3 μM in these four cell lines with partial reversal at concentrations of 0.03 to 0.1 μM (Figure 2

). In the absence of TGFα, ZD 1839 inhibited growth of PE01 cells at concentrations ⩾0.03 μM, SKOV-3 cells at concentrations ⩾0.3 μM and PE04 cells at 3 μM (Figure 2). The A2780 cell line showed no growth stimulation by TGFα and minimal growth effects (0, 6 and 19% inhibition at 0.3, 1 and 3 μM ZD 1839 respectively) in response to ZD 1839 (data not shown). Growth inhibition in the other cell lines was up to approximately 50% of control cell number and may reflect the presence of endogenous TGFα or other EGFR-activating ligands. To test this possibility of autocrine regulation, the presence of TGFα, EGF and amphiregulin in the cell lines were sought by the use of RT–PCR. All four TGFα-responsive cell lines were found to express mRNAs for these three growth factors, indicating the potential for autocrine regulation, whereas A2780 was negative (Figure 3

).

### Inhibition of tyrosine phosphorylation of the EGFR by ZD 1839

**Figure 4:**
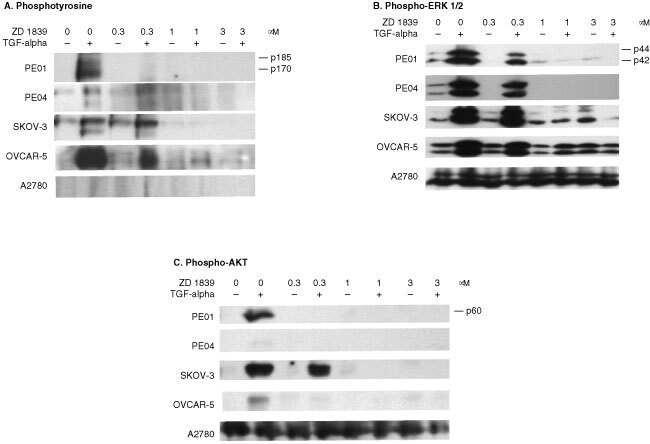
Western blot of ZD 1839 treated cell lines using (**A**) the anti-phosphotyrosine PY20 antibody, (**B**) the phospho-ERK NEB 9101 antibody and (**C**) the phospho-AKT^S473^ NEB 9271 antibody. Blots are shown for the PE01, PE04, SKOV-3, OVCAR-5 and A2780 cell lines. ZD 1839 was added 30 min prior to TGFα (1 nM) addition and lysates were collected after 15 min exposure to TGFα. Amounts of protein loaded were 75 μg lane^−1^ for the phosphotyrosine gel and 35 μg for the phospho-ERK and phospho-AKT gels. Exposure times varied from 20 s to 2 min but were the same for each antibody.

The cell lines were pre-treated with ZD 1839 for 30 min before a 15 min incubation in the presence or absence of 1 nM TGFα. TGFα increased tyrosine phosphorylation of both the EGFR and ErbB2 in the four responsive cell lines (PEO1, PEO4, SKOV-3 and OVCAR-5) consistent with ligand activation via the EGFR and heterodimerization with ErbB2 (Figure 4A

). A2780 showed no band for either EGFR or ErbB2. The identity of bands had been confirmed in previous experiments using antibodies specific for the EGFR and erbB2 (data not shown). At concentrations of ZD 1839 >0.3 μM, the EGFR phosphorylation was completely blocked in all cell lines except OVCAR-5 in which EGFR phosphorylation was blocked at concentrations of ZD 1839 >1 μM. The ErbB2 phosphorylation was also decreased but to a lesser degree in all four responsive lines (Figure 4A). In the absence of TGFα, only ErbB2 phosphorylation was observed in untreated cells (Figure 4A). This was also decreased after addition of ZD 1839 but only at higher concentrations of inhibitor.

### Inhibition of the ERK and PI3-kinase pathways by ZD 1839

Lysates of the five cell lines were also probed with antibodies recognizing phospho-ERK, a member of the MAP kinase cascade, and to phospho-AKT^S473^, downstream of PI-3 kinase. TGFα increased phosphorylation of both ERK 1 and 2 (p44 and p42) in the four responsive cell lines, but not in A2780 (Figure 4B). ZD 1839 blocked ERK phosphorylation at 1 and 3 μM, reflecting the results for EGFR and erbB2 tyrosine phosphorylation (Figure 4A). A similar result was obtained for phospho-AKT, with the four responsive lines showing phosphorylation induced by TGFα, which was blocked in all but the SKOV-3 cell line at the lower concentration of 0.3 μM ZD 1839. Both phospho-ERK and phospho-AKT were elevated at basal conditions in A2780 cells compared to the other cell lines and neither TGFα nor ZD 1839 had any effect on expression (Figure 4B,C).

### Inhibition of the PE04 ovarian cancer xenograft by ZD 1839

**Figure 5:**
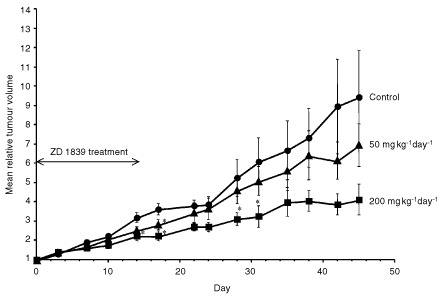
Effect of ZD 1839 on the growth of established PE04 xenograft grown in nude mice. Groups of nude mice received either vehicle only or daily oral treatment of ZD 1839 at either 200 mg kg^−1^ day^−1^ or 50 mg kg^−1^ day^−1^. Points shown are mean values for groups of 5–8 tumours. Error bars= standard deviation. Statistically significant differences from control: *P<0.05, Student *t*-test.

The ability of ZD 1839 to inhibit growth *in vivo* was assessed using the PE04 ovarian cancer model. Previous xenograft studies have used ZD 1839 at doses of 150–250 mg kg^−1^ day^−1^ (Ciardiello *et al*, 2000; Sirotnak *et al*, 2000). ZD 1839 when given orally at 200 mg kg^−1^ day^−1^ for 14 days produced >50% inhibition of control growth at the end of the treatment period, and this effect persisted for a further 31 days, at which point the experiment was terminated (Figure 5

). A lower dose, 50 mg kg^−1^ day^−1^ produced a small effect with statistical significance at day 17 (i.e. immediately after the full treatment course) but this effect did not persist (Figure 5). The effect of continued treatment at either dose was not evaluated. At 50 mg kg^−1^ day^−1^ there was no mean body weight loss compared to control at end of treatment (day 14) and after a further 14 days (day 28) while at 200 mg kg^−1^ day^−1^ there was a small mean weight loss (9.8±1.6% on day 14 and 10.9±4.5% on day 28).

## DISCUSSION

Strategies targeting the EGFR may well have therapeutic potential in ovarian cancer as the receptor is frequently overexpressed in this disease and patients with tumours expressing high levels of EGFR tend to have a poor prognosis. ZD 1839 is one of the latest inhibitors developed against this receptor and, in addition to being both potent and selective, it is orally active. This is the first detailed study of ZD 1839 in a range of ovarian cancer cell lines and our results extend the previous observations of broad spectrum activity for this agent against a range of xenograft types including cancers of the lung, colon, prostate and vulva (Ciardiello *et al*, 2000; Sirotnak *et al*, 2000). We observed good reversal of EGFR tyrosine phosphorylation produced by TGFα activation in ovarian cancer cell lines and this was consistent with growth inhibition at these concentrations. In the absence of added TGFα, the cell lines were shown to produce mRNAs for several EGFR-activating ligands indicating the strong possibility of autocrine regulation. Under these conditions, ZD 1839 partially inhibited growth of several of the cell lines consistent with blockade of activation via this endogenous expression. Extension into a xenograft model confirmed the activity of the drug given orally to the PE04 ovarian cancer *in vivo*. We have previously investigated the activity of another anilinoquinazoline, ZM 252868 (PD 153035) which had similar *in vitro* properties to those of ZD 1839; however its activity *in vivo* was limited and while it demonstrated transient reduction of EGFR tyrosine phosphorylation in A431 xenografts, this was insufficient to produce *in vivo* growth inhibition (Kunkel *et al*, 1996). ZD 1839 possesses significant *in vivo* activity and is now being evaluated in clinical trials.

Western blot probing for tyrosine phosphorylation confirmed that ZD 1839 was acting as predicted, with the inhibitor targeting the EGFR and having a secondary effect on ErbB2 phosphorylation. The latter is likely to be due to inhibition of heterodimer phosphorylation. Furthermore, the same concentrations of ZD 1839 shown to inhibit receptor activation were associated with growth inhibition. Phosphorylation events in the downstream effector molecules of the MAP kinase (phospho-ERK) and PI-3 kinase (phospho-AKT) pathways revealed some of the complexity involved with signal transduction from these receptors. There was a striking similarity between phosphorylation and hence activation of the ErbB receptors in response to both TGFα stimulation and receptor blockade, and events in the downstream signalling cascades. Phosphorylation profiles of ERK and AKT over the concentration ranges closely resembled that of the two ErbB receptors over the concentration ranges and was consistent with both signalling cascades being induced as a consequence of receptor activation. It is likely that signalling via one or both of the pathways leads to the mitogenic effect seen upon TGFα stimulation, and hence its loss upon blockade.

In addition to the strategy investigated here, we have previously shown that antibody blockade of the EGFR produces similar growth reversing effects in the PE01 cell line (Simpson *et al*, 1998). The use of antibodies to target the EGFR is well advanced in clinical trials and a humanized/mouse chimeric monoclonal antibody 225 is undergoing evaluation.

Promising clinical results in head and neck cancer have been obtained when EGFR inhibition has been combined with radiotherapy or chemotherapy (Ezekial *et al*, 1999; Mendelsohn *et al*, 1999). It is likely that other EGFR targeting strategies might optimally be used in combination with other drugs. To this end, combination of ZD 1839 with a range of cytotoxics have already produced interesting results in a range of tumour types *in vitro* and *in vivo* (Ciardiello *et al*, 2000; Sirotnak *et al*, 2000). Among the cell lines tested *in vitro* was the OVCAR-3 ovarian cancer cell line; supra-additive effects were observed for ZD 1839 in combination with cisplatin, carboplatin paclitaxel and docetaxel (Ciardiello *et al*, 2000).

In the clinical setting, it will be important to define tumours whose growth is dependent on the EGFR and which are being driven by activating ligands such as TGFα and EGF. It is apparent from these data that levels of EGFR protein expression are not simply associated with magnitude of ligand response as even cell lines with low levels of EGFR can show a marked stimulation when treated with TGFα. However, tyrosine phosphorylation of the EGFR in clinical specimens of ovarian cancer can be readily identified and it seems likely that those tumours in which activation is found represent the target population for these agents.

In conclusion, these data indicate that the EGFR-targeted tyrosine kinase inhibitor ZD 1839 can inhibit growth of ovarian cancer cells *in vitro* and *in vivo*, consistent with inhibition of tyrosine phosphorylation at the EGFR. These results lends further support to the view that targeting the EGFR in ovarian cancer could have therapeutic value.
